# Real-Time PyMOL Visualization for Rosetta and PyRosetta

**DOI:** 10.1371/journal.pone.0021931

**Published:** 2011-08-16

**Authors:** Evan H. Baugh, Sergey Lyskov, Brian D. Weitzner, Jeffrey J. Gray

**Affiliations:** 1 Department of Chemical and Biomolecular Engineering, The Johns Hopkins University, Baltimore, Maryland, United States of America; 2 Program in Molecular Biophysics, The Johns Hopkins University, Baltimore, Maryland, United States of America; University of South Florida, United States of America

## Abstract

Computational structure prediction and design of proteins and protein-protein complexes have long been inaccessible to those not directly involved in the field. A key missing component has been the ability to visualize the progress of calculations to better understand them. Rosetta is one simulation suite that would benefit from a robust real-time visualization solution. Several tools exist for the sole purpose of visualizing biomolecules; one of the most popular tools, PyMOL (Schrödinger), is a powerful, highly extensible, user friendly, and attractive package. Integrating Rosetta and PyMOL directly has many technical and logistical obstacles inhibiting usage. To circumvent these issues, we developed a novel solution based on transmitting biomolecular structure and energy information via UDP sockets. Rosetta and PyMOL run as separate processes, thereby avoiding many technical obstacles while visualizing information on-demand in real-time. When Rosetta detects changes in the structure of a protein, new coordinates are sent over a UDP network socket to a PyMOL instance running a UDP socket listener. PyMOL then interprets and displays the molecule. This implementation also allows remote execution of Rosetta. When combined with PyRosetta, this visualization solution provides an interactive environment for protein structure prediction and design.

## Introduction

The Rosetta software suite, an object-oriented protein structure prediction tool [1], can effectively perform protein structure prediction and design [2] [3]. Common tasks such as design, docking, and folding produce text files containing the Cartesian coordinates of each atom of the protein. Molecular visualization tools are used later to interpret the output [4]. Thus, visualization is typically separated from Rosetta. Output structures could be viewed after a simulation, but not during a calculation or protocol. Real-time structural visualization would facilitate development of new methods and make Rosetta more accessible to new users.

Rosetta protocols are useful to a broad range of scientists, but many protocols are complex. Scientists who are not trained in computation can become frustrated with Rosetta's steep learning curve. Two new interfaces to Rosetta were built to bridge this gap. PyRosetta [5] is a Python-based interface to Rosetta objects and protocols enabling users to easily develop custom algorithms and to explore Rosetta through the Python interpreter. RosettaScripts [6] provides an XML-scriptable interface to Rosetta allowing users to design custom algorithms. PyRosetta and RosettaScripts have successfully lowered the barrier to learn Rosetta by providing an interactive and scriptable layer. To further improve access to Rosetta, the next step is to provide an intuitive way of observing protocols with elegant informative graphics.

Several visualization tools have been previously employed which were ideal for some users, but lacked universal appeal. FoldIt [7] is a Rosetta-based interactive video game with rich graphics intended for end users to explore protein structures and energetics. It does not allow the loading of arbitrary proteins, lacks access to many Rosetta features, and removes the user from the underlying code, so it is not appropriate as a development environment. Rosetta graphics mode is an undocumented optional feature of Rosetta protocols used by the internal Rosetta community for error checking. Running a protocol in graphics mode produces an image of the manipulated structure, enabling the viewer to see changes, but not to interact with them. Using graphics mode requires knowledge of the Rosetta build system, limiting it to advanced users and developers. PyMOL [8] has a very large user base, is not restricted to the visualization of Rosetta output, and is maintained by Schrödinger, but viewing Rosetta structures in PyMOL requires outputting the atomic coordinates to a PDB file and then loading it into PyMOL. A new software plug-in called ePMV [9] unifies and visualizes molecular and cellular simulations including Rosetta.

In this paper, we describe a new solution that allows real-time visualization of Rosetta via PyMOL using UDP network sockets. The versatile communication presents structural and energetic information on demand making Rosetta more accessible to all users.

## Materials and Methods

**Figure 1:**
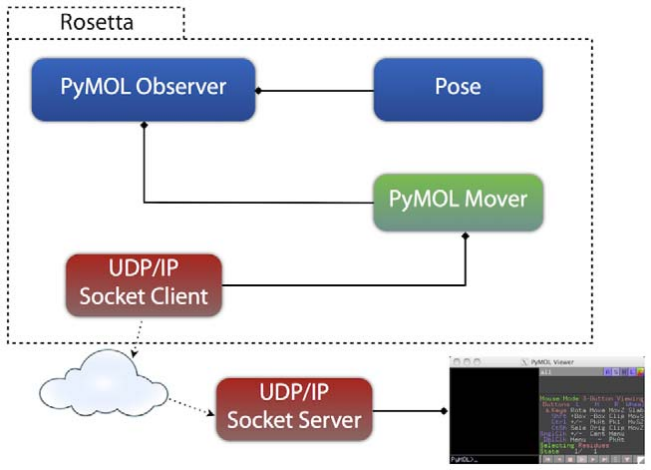
Rosetta-PyMOL network communication. Rosetta transmits data through the PyMOL_Mover's UDP/IP socket client to an IP address. Dotted arrows represent network communication and diamonds represent composition (e.g. the PyMOL Observer contains a PyMOL Mover and an [owning pointer to a] Pose). The PyMOL Observer monitors changes in a Pose and uses the PyMOL Mover to transmit this information to PyMOL. The UDP/IP socket server running in PyMOL listens for network traffic and translates appropriate packets. Once the data is translated, PyMOL displays biomolecular structures.

Since both PyRosetta and PyMOL are Python-based, a possible visualization solution is to run PyRosetta from within the PyMOL interpreter window. However, Python is notorious for compatibility issues between different versions, and thus technical obstacles prevent a robust implementation of this idea. We developed a completely different approach: use a network protocol to transfer data from PyRosetta to PyMOL (Figure 1). Both TCP/IP and UDP/IP implementations were viable. The latter was chosen because it does not require implicit “hand-shaking” and tolerates lost transmissions, making it more robust. Both programs can transmit, open or close, and even crash without dependence on the other. Accordingly, since the UDP protocol does not guarantee reliability, data must be validated for proper delivery. Restrictions on packet size also forces large packets to be split and sent separately.

A Python script running in PyMOL provides a server that listens for incoming UDP/IP traffic. Each packet (Table 1) is recognized by this server, and specific actions, such as coloring or model construction, occur when the server receives a complete packet. Accumulated packets could be fragments of a large data transfer, and the desired action happens only once all pieces are assembled. Data from different PyRosetta instances and errors in network communication can confuse interpretation, so any incomplete information received is held in memory briefly and later, if necessary, released in the rare case that a message is aborted.

**Table 1:** Packet structure.

Size	Description
(bytes)	
16	UUID of the sender
4	Unique packet id for this sender/packet
4	Sub-packet id
4	Total number of sub-packets
8	Type of packet (8 byte string) e.g.: “PDB.bz2”, “Ener.bz2”, “PDB.gzip”, or “Ene.gzip”
1	Flag indicating whether PyMOL should load the PDB into the current state or the next state
1+N	Length of model name in bytes + name (N)
1+N	Length of energy name in bytes + name (N)
X	Compressed packet data, a PDB file string or energy float array compressed with bz2 or gzip

Rosetta's preexisting Mover architecture is suited to manage the communication because a Mover can accept a pose (Rosetta's container for a molecular object) and perform operations on the pose data. Using the Mover architecture is intuitive for Rosetta users and allows the PyMOL_Mover to interact with other Rosetta objects. The PyMOL_Mover is a simple network client which sends protein structure and energy information when its apply() method is invoked. Rosetta builds each data packet in a specific order (Table 1) for recognition by the PyMOL listener. We considered sending Python commands directly to PyMOL, however, restricting communication to specific packets reduces security risks since PyMOL only understands and performs a small set of commands.

### Software Availability

The PyMOL script to interpret Rosetta results is included with Rosetta (/mini/src/python/bindings) and PyRosetta (root directory). Rosetta is available for download at http://www.rosettacommons.org/. PyRosetta is available for download at http://www.pyrosetta.org. The PyMOL_Mover is contained in Rosetta beginning with release 3.2 (revision 37389). Both programs have free licenses for academic and nonprofit institutions. PyMOL mus be obtained from Schrödinger (the PyMOL_ Mover has been successfully tested with PyMOL versions 1.0, 1.2, and 1.3).

### Protocol Capture

Proper usage requires (1) a sender, Rosetta or PyRosetta, with the PyMOL_Mover; and (2) a listener, PyMOL, with the listener script. We first demonstrate how to setup the PyMOL server features. Then we present examples of PyMOL_Mover usage with PyRosetta. Finally, we explain how to incorporate the PyMOL_ Mover into Rosetta protocols.

#### PyMOL Protocol

After loading PyMOL, use the command line (e.g. Tk Window Upper Command Line) to run the script PyMOLPyRosettaServer.py to start the listener:

run /path/to/PyRosetta/PyMOLPyRosettaServer.py

run/path/to/Rosetta/mini/src/python/bindings/PyMOLPyRosettaServer.py

If desired, Rosetta and PyMOL can run on different computers. In this case, PyMOL must link to a new IP address using the start_rosetta_server command with the desired IP address and port number as arguments. A single instance of PyMOL can listen to multiple IP addresses or ports by invoking start_rosetta_server multiple times with different arguments.

start_rosetta_server 125.1.3.37, 9001

#### PyRosetta Protocol

In Rosetta and PyRosetta, a macromolecule (and information on a single conformation) is stored in a pose object. To explore the PyMOL_Mover, a user will start PyRosetta and create a pose object from a PDB file.

from rosetta import*

init()

pose = Pose() pose = pose_from_pdb(pose,“test_dock.pdb”)

When the PyMOL_Mover is applied to a pose, the pose coordinate data is sent to PyMOL rendering an image of the structure.

pymover = PyMOL_Mover()

pymover.apply(pose)

The PyMOL_Mover can be applied repeatedly throughout a custom protocol to update coordinates in the PyMOL window. In this way, a user can view any intermediate step throughout a structure prediction or design protocol.

**Figure 2:**
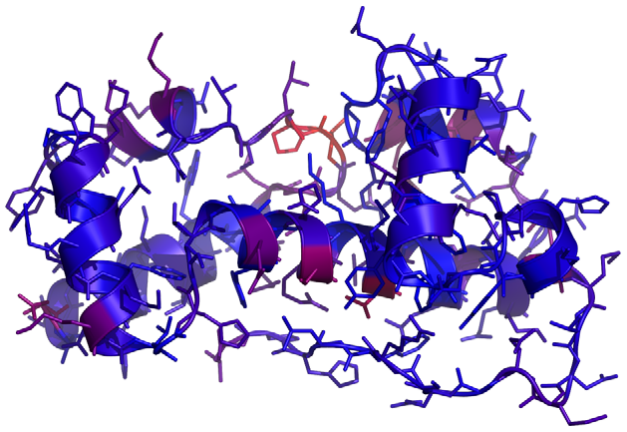
Example PyMOL structure. An example of the crystal structure in decoy test_in.pdb, available with Rosetta, as output by the PyMOL_Mover. The residues are colored based on their energy evaluation with the standard Rosetta score function ranging from red (high energy) to blue (low energy). One loop region scores noticeably higher than other residues in the protein.

To color the loaded structure based on the relative residue energies, a PyMOL_Mover.send_energy method is provided. The pose must have been scored before applying the PyMOL_Mover. Using the PDB file test_in.pdb (provided in File S1) with these commands demonstrates Rosetta's ability to recognize high-energy protein regions (Figure 2).

scorefxn = create_score_function(“standard”)

scorefxn(pose)

pymover.apply(pose)

pymover.send_energy(pose)

The default color spectrum spans from blue (low score/favorable energy) to red (high score/unfavorable energy). Rosetta scores are determined using a weighted sum of various score terms. The send_energy method can accept the name of any score term and color residues based on this term as long as it is used in the current score function (i.e. a non-zero weight). This example colors the residues based on the value of their (all-atom) Van der Waals attractive energy:

pymover.send_energy(pose,“fa_atr”)

The PyMOL_Mover can also automatically color residues by energy every time the coordinates are transmitted by setting the update_energy option to true.

pymover.update_energy = True

pymover.apply(pose)

The PyMOL_Mover can transmit a specific score term with the coordinates during an apply() command if the energy_type option is set and update_energy is true. This example colors the residues based on the value of their (all-atom) solvation energy every time the PyMOL_Mover is applied:

pymover.update_energy = True

pymover.energy_type = “fa_sol”

pymover.apply(pose)

To send PyMOL_Mover output to a PyMOL instance on a different computer, the PyMOL_Mover.link options can be modified:

pymover.link.udp_ip = “125.1.3.37”

pymover.link.udp_port = 9001

If the PyMOL_Mover's keep_history option is set true, PyMOL will load structures with the same name (pose.pdb_info().name()) into successive states.

pymover.keep_history = True

pymover.apply(pose)

other_mover.apply(pose)

pymover.apply(pose)

In PyMOL, the object states can be viewed as a movie which reveals the Rosetta protocol actions. The output can easily be converted into a movie using PyMOL's frame and movie building features.

In the previous examples, PyMOL updates have been manually transmitted using PyMOL_Mover.apply(). The PyMOL_Observer object automatically monitors a pose conformation and applies its own PyMOL_Mover every time the pose coordinates are changed. Since a PyMOL_Observer has a PyMOL_Mover, all of the PyMOL_Mover options are available.

pyobs = PyMOL_Observer()

pyobs.add_observer(pose)

pyobs.pymol.update_energy = True

A sample Python script demonstrating how to use the PyMOL_Mover for making movies is PyRosettaDock_Movie.py in File S1. File S1 also includes a more in-depth tutorial script, PyMOL_demo.py, and a copy of the listener, PyMOLPyRosettaServer.py.

#### Rosetta Protocols

All PyRosetta features can also be accessed in the Rosetta C++ code by using appropriate C++ syntax and changes including the namespace.

#include <protocols/moves/PyMOL_Mover.hh>

core::protocols::moves::AddPyMOLObserver(pose)

## Results and Discussion

Rosetta output to PyMOL is now available on-demand and continuously. Two separate objects have been implemented with Rosetta's object-oriented framework. The PyMOL_Mover object sends data to PyMOL and allows the user to observe visual output on-demand. This implementation is ideal for interactive environments and allows instant feedback from custom processes. New users can now experiment with Rosetta structures and protocols while immediately viewing their changes.

A second object, the PyMOL_Observer, can be attached to a Rosetta biomolecule (pose) to monitor all structural changes. When a coordinate is updated, the observer uses an embedded PyMOL_Mover to transmit the new structure to PyMOL. This implementation is ideal for elaborate scripts where manual management of output is prohibitive. Furthermore, the observer can be added to previously developed protocols, providing visual output with minimal modification. One limitation is that continuous PyMOL packet construction can slow Rosetta calculations. To mitigate this delay, a user can optionally output coordinates at particular time or coordinate-update intervals (see documentation).

In addition to being a useful tool to display protein structures, PyMOL is well suited for the production of informative movies. Rosetta simulations are often presented visually to demonstrate or explain the principles underlying Rosetta algorithms. Previously, making movies of Rosetta protocols required significant work. When sending data to PyMOL, the user may simply retain output history to produce PyMOL movies. The history feature also allows the user to inspect protocols that are otherwise inaccessible.

The PyMOL_Mover can also color residues allowing easy recognition of energetic features and design anomalies. Although only Rosetta score values are currently supported, any relevant data calculated in Rosetta may be sent to PyMOL using this interface. Additional features can be added easily for the visualization of other Rosetta data as long as these data packets carry specific tags for PyMOL to translate.

The UDP/IP client utilization has several advantages. PyMOL can be customized to receive information from multiple instances of Rosetta. Communication is not limited by the operating environments of either the sender or the receiver. Data can be successfully interpreted between different operating systems (e.g. Linux to Windows), Python versions, and even buildings. A single Rosetta process can transmit data to multiple IP addresses or ports using multiple PyMOL_Movers, allowing visualization on multiple computers. This versatility reduces communication errors and allows users to share information easily. Running each program separately prevents either from losing focus; Rosetta performs simulations and calculations while PyMOL performs visualization.

The ability to view processes in real-time can easily produce relevant examples for presentations and demonstrations. The UDP solution is inherently unrestricted and is potentially applicable to numerous other applications. Additional protein modeling software such as the SWISS-MODEL server [10], CHARMM [11], and NAMD [12] could also output to PyMOL with socket export. Similarly, other graphical software such VMD [13], RasMol [14], Deep View [15], Kinemage [16] could interpret and display data packets sent from Rosetta by listening to output from a socket. If such practices become widespread, communication using program independent packet structures could lead to a standardized macromolecular visualization markup language.

## Supporting Information

File S1 **A summary of the supplemental files contained in the compressed directory File S1, including the PyMOL listener, sample scripts with their data files, and a representative movie of the script output.** README: a brief tutorial to the PyMOL_Mover and these scripts. PyRosettaDock.mp4: a PyMOL movie of Rosetta's Docking Protocol. input_files/: PDB files used in the demo scripts. test_dock.pdb a modified 1ACB [17] PDB file used in PyRosettaDock_Movie.py. test_in.pdb the decoy file PDB used in PyMOL_demo.py. scripts/: sample PyRosetta syntax for using the PyMOL_Mover. PyMOLPyRosettaServer.py listener script for PyMOL. PyRosettaDock_Movie.py sample protocol to create a docking movie. PyMOL_demo.py numerous PyRosetta sample commands with the PyMOL_Mover.
(TAR.BZ2)

## References

[1] Leaver-Fay A, Tyka M, Lewis SM, Lange OF, Thompson J (2011). ROSETTA3: An objectoriented software suite for the simulation and design of macromolecules.. Methods in Enzymology.

[2] Das R, Baker D (2008). Macromolecular modeling with Rosetta.. Biochemistry.

[3] Kaufmann KW, Lemmon GH, DeLuca SL, Sheehan JH, Meiler J (2010). Practically useful: What the Rosetta protein modeling suite can do for you.. Biochemistry.

[4] O'Donoghue SI, Goodsell DS, Frangakis AS, Jossinet F, Laskowski RA (2010). Visualization of macromolecular structures.. Nature Methods.

[5] Chaudhury S, Lyskov S, Gray JJ (2010). PyRosetta: a script-based interface for implementing molecular modeling algorithms using Rosetta.. Bioinformatics.

[6] Fleishman SJ, Leaver-Fay A, Corn JE, Khare SD, Koga N (2011). RosettaScripts: an XMLlike interface to the Rosetta macromolecular modeling suite.. Plos ONE.

[7] Cooper S, Khatib F, Treuille A, Barbero J, Lee J (2010). Predicting protein structures with a multiplayer online game.. Nature.

[8] DeLano WL (2002). PyMOL molecular graphics system.. http://www.pymol.org.

[9] Johnson GT, Autin L, Goodsell DS, Sanner MF, Olson AJ (2011). ePMV Embeds Molecular Modeling into Professional Animation Software Environments.. Structure.

[10] Schwede T, Kopp J, Guex N, Peitsch MC (2003). SWISS-MODEL: An automated protein homologymodeling server.. Nucleic Acids Research.

[11] Brooks BR, Bruccoleri RE, Olafson BD, States DJ, Swaminathan S (1983). CHARMM: A program for macromolecular energy, minimization, and dynamics calculations.. Computational Chemistry.

[12] Wang Y, Harrison CB, Schulten K, McCammon JA (2011). Implementation of accelerated molecular dynamics in NAMD.. Computational Science and Discovery.

[13] Humphrey W, Dalke A, Schulten K (1996). VMD: Visual molecular dynamics.. Molecular Graphics.

[14] Sayle RA, Milner-White EJ (1995). RasMol: Biomolecular graphics for all.. Trends in Biochemical Sciences.

[15] Guex N, Peitsch MC (1997). SWISS-MODEL and the Swiss-PDB Viewer: An environment for comparative protein modeling.. Electrophoresis.

[16] Richardson DC, Richardson JS (1992). The Kinemage: A tool for scientific communication.. Protein Science.

[17] Frigerioa F, Codab A, Puglieseb L, Lionettib C, Menegattic E (1992). Crystal and molecular structure of the bovine *α*-chymotrypsin-eglin c complex at 2.0 Å resolution.. Journal of Molecular Biology.

